# A Short Video Classification Framework Based on Cross-Modal Fusion

**DOI:** 10.3390/s23208425

**Published:** 2023-10-12

**Authors:** Nuo Pang, Songlin Guo, Ming Yan, Chien Aun Chan

**Affiliations:** 1 School of Design, Dalian University of Science and Technology, Dalian 116052, China; pangnuo@dlust.edu.cn; 2 School of Information and Communications Engineering, Communication University of China, Beijing 100024, China; 3 Insta-Wireless, Notting Hill, VIC 3168, Australia; chienac@unimelb.edu.au; 4 Department of Electrical and Electronic Engineering, The University of Melbourne, Parkville, VIC 3010, Australia

**Keywords:** video classification, cross-modal fusion, video features, text features, Timesformer

## Abstract

The explosive growth of online short videos has brought great challenges to the efficient management of video content classification, retrieval, and recommendation. Video features for video management can be extracted from video image frames by various algorithms, and they have been proven to be effective in the video classification of sensor systems. However, frame-by-frame processing of video image frames not only requires huge computing power, but also classification algorithms based on a single modality of video features cannot meet the accuracy requirements in specific scenarios. In response to these concerns, we introduce a short video categorization architecture centered around cross-modal fusion in visual sensor systems which jointly utilizes video features and text features to classify short videos, avoiding processing a large number of image frames during classification. Firstly, the image space is extended to three-dimensional space–time by a self-attention mechanism, and a series of patches are extracted from a single image frame. Each patch is linearly mapped into the embedding layer of the Timesformer network and augmented with positional information to extract video features. Second, the text features of subtitles are extracted through the bidirectional encoder representation from the Transformers (BERT) pre-training model. Finally, cross-modal fusion is performed based on the extracted video and text features, resulting in improved accuracy for short video classification tasks. The outcomes of our experiments showcase a substantial superiority of our introduced classification framework compared to alternative baseline video classification methodologies. This framework can be applied in sensor systems for potential video classification.

## 1. Introduction

In the past few years, the emergence of short video applications has exploded, such as Tiktok, YouTube Shorts, Likee, Bilibili. Most of the short videos in these video applications are tagged when they are released, enabling users to browse videos by category and search within a certain category [1]. These short videos are typically characterized by their brief duration, diverse content, and a wide range of topics. However, the exponential increase in the number of short videos poses significant challenges in terms of effectively classifying and managing this vast video content.

At present, the application of deep learning methods in the classification of violent videos [2] and social media videos [3,4] has achieved good results. However, due to the unique characteristics of short videos, such as the short duration, large amount, and many spliced contents, it is still a difficult task to classify short videos. Research on short video classification predominantly employs single-modal approaches which utilize either visual or textual features for classification. Visual feature extraction involves the extraction of image features from video frames, encompassing attributes such as color histograms, texture features, and shape characteristics. Studies have demonstrated the high efficacy of visual features in short video classification, as they facilitate the capture of visual information within the videos, including objects, scenes, and actions. Traditional techniques for visual feature extraction comprise the histogram of oriented gradient (HOG) [5], histogram of flow (HOF) [6], and motion boundary histograms (MBH) [7]. To leverage the complementarity of these three feature types and enhance the representational capacity of video features, researchers have introduced the dense trajectories (DT) algorithm [8] and its improved variant, the improved dense trajectories (IDT) [9]. These algorithms are both based on decision tree classification approaches, and utilize HOG descriptors as image features derived by statistically analyzing the gradient information of images.

In addition to visual features, textual features are also widely employed in the field of short video classification. These textual features typically originate from metadata information such as video titles, descriptions, and tags. The advantage of textual features lies in their ability to provide semantic information about the video content, thereby enhancing classification accuracy. Researchers have developed various methods for extracting textual features, including those based on traditional natural language processing techniques such as the bag of visual words (BOVW) model [10], as well as deep learning-based methods such as recurrent neural networks (RNNs) and attention mechanisms. The core idea of BOVW is to represent images as a collection of visual words and use the frequency of word occurrences as the image’s feature vector [11]. Firstly, local features are extracted from the image, such as scale-invariant feature transform (SIFT) [12], local binary patterns (LBP) [13], color histograms, etc. Subsequently, all local features are clustered into several clusters, with each cluster corresponding to a visual word. The frequency of each word’s occurrence is computed, and finally, this feature representation is employed for tasks such as training classifiers. The convolutional neural networks (CNNs) primarily decompose videos into a sequence of frames and then extract features from each frame through multiple layers of convolutional and pooling operations. These extracted features from all frames are aggregated and used for classification with the assistance of a classifier.

However, at present, users upload short videos with great randomness and divergence, and users’ understanding of video categories generally varies and there is more and more false information, resulting in inconsistent categories marked by users [14]. This inconsistency not only affects the accuracy of the search and recommendation results of video content categories, but also in the face of these challenges, users are more inclined to make subjective judgments through visual content to meet their personal needs. In addition, there is a significant difference between video content features and hashtag text features. It is difficult to match accurate hashtags to meet users’ content consumption needs due to insufficient video text information in the method of searching for tags with the same text in videos [15,16]. Moreover, some videos usually do not contain classification information, and video feature analysis is mainly based on understanding visual image information, but lacks text semantic mining, resulting in an underutilization of semantic information [17,18].

Thus, short video classification is essential to determine the category of a video so that videos without user-labeled categories can also be organized in the same way as videos with category labels. A distinct video classification framework is introduced herein which leverages both textual and visual features in a new way. We bring together visual features obtained from the training dataset with text features extracted from subtitles across modalities, and integrate them into joint features for downstream classification tasks. Specifically, the text feature uses the bidirectional encoder representation from the Transformers (BERT) pre-training model, adds context using the attention mechanism, and solves the parallel calculation between sentences [19,20,21]. Video features are extended from image space to spatio-temporal three-dimensional volume through a self-attention mechanism, which treats video as a series of patches extracted from a single frame. Like the vision Transformer (VIT), each patch undergoes linear mapping into an embedding, to which positional data are subsequently incorporated [22]. The proposed framework uses textual and visual features to classify short videos, which improves the accuracy of short video classification. The related techniques can be applied in sensor systems for potential video classification, so the subject of this paper belongs to data fusion and analysis in sensor systems. The main contributions are as summarized below:

1. We propose an improved hierarchical clustering approach for keyframe extraction. Unlike traditional keyframe extraction algorithms, hierarchical clustering does not require a predefined number of keyframes to be extracted. Instead, it adaptively determines which frames are keyframes through clustering to offer greater flexibility. This method is capable of preserving essential information from the video while effectively reducing redundant frames, resulting in more representative extracted keyframes.
2. We investigate the extraction methods of visual features and textual features within videos. The method of combining visual information and text information for video classification is used in this paper. The visual information is first processed by the key frame extraction method to divide the video into multiple images representing the main content. The pre-trained Timesformer network is used for feature extraction to obtain the visual features of the video. At the same time, the text information is also extracted by the fine-tuning-based method in BERT. Finally, these two kinds of features are fused by the feature aggregation algorithm for video classification.
3. We propose a cross-modal fusion short video classification (CFVC) framework. This framework utilizes text features and visual features in a new way, combining the integration of visual attributes extracted from the training dataset and text features extracted from subtitles to achieve cross-modal fusion and integrate them into joint features for downstream classification tasks.

The subsequent sections of this paper are structured as follows. Section 2 summarizes the existing work related to this paper. Section 3 introduces the proposed cross-modal fusion short video classification framework. Section 4 evaluates the proposed framework through experiments. Section 5 concludes our work.

## 2. Related Work

**Figure 1:**
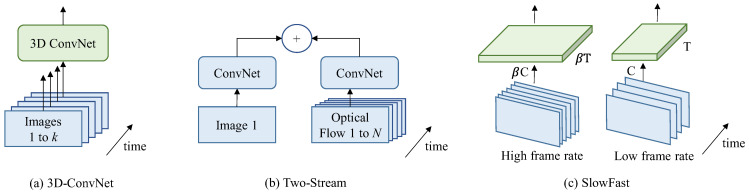
Different types of video classification architectures (**a**) 3D-ConvNet, (**b**) Two-Stream and (**c**) SlowFast.

Despite considerable progress having been achieved for image representation architectures over recent years, the realm of video architecture remains devoid of a distinctly defined forefront structure. The current main video classification architectures are shown in Figure 1, where *k* represents the count of frames within a video, and *N* represents a subset of adjacent frames of the video. The main differences between these frameworks are: (1) The first differentiation lies in determining whether the convolution and layer operators utilize 2D (image-based) or 3D (video-based) kernels [23,24,25]. (2) Another key variation involves the nature of the input provided to the network. This can be limited to just an RGB video or expanded to encompass both an RGB video and pre-computed optical flow [26,27,28]. (3) In the context of 2D convolutions, a significant consideration is how information propagates across frames. This can be achieved through the incorporation of temporary recurrent layers such as SlowFast or the application of feature aggregation over time [29,30,31].

### 2.1. I3D Networks

Traditional 2D convolutional neural networks have been a huge success in tasks such as image classification, but there are some challenges in video classification tasks. To make the most of temporal information and motion features in videos, researchers proposed a variety of three-dimensional convolutional network (3D ConvNet) models as shown in Figure 1a [23]. The inflated 3D ConvNet (I3D) model is extended on the basis of a two-dimensional convolutional network. Specifically, it constructs a three-dimensional convolutional network structure by copying and filling the weights of the pre-trained two-dimensional convolutional network in the time dimension [24]. This approach enables the I3D model to simultaneously process features in both spatial and temporal dimensions, thereby better capturing dynamic information in videos. To efficiently train the I3D model, two strategies are adopted: pre-training of the second-rate network and multi-scale cropping [25]. First, by pre-training on a large-scale video dataset, the I3D model can learn rich visual features. Then, it is fine-tuned on the dataset of the target task to improve its performance on the specific task. In addition, to take advantage of the spatio-temporal information in the video, a multi-scale cropping strategy is also introduced, which is trained by extracting multiple cropped segments of different scales from the video. Applications of I3D models have achieved remarkable results in several video understanding tasks.

### 2.2. Two-Stream Networks

Simulations of high-level changes can be achieved by the long short-term memory (LSTM) networks based on features extracted from the final convolutional layer, but the capturing of essential fine-grained low-level motion, pivotal in numerous scenarios, might be hindered [26]. Training also incurs significant costs, given the necessity for the network to be unrolled across multiple frames to facilitate time-based backpropagation. An enhanced methodology entails the modeling of brief temporal video snapshots, achieved by combining forecasts originating from an individual RGB frame and a compilation of 10 externally generated optical flow frames. This is subsequently followed by the traversal of two iterations of an ImageNet-pre-trained ConvNet [27]. An adapted input convolutional layer is integrated within the two-stream architecture, boasting double the number of input channels in comparison to the frames within the stream as shown in Figure 1b. During the testing phase, numerous video snapshots are sampled and subsequently aggregated to yield action predictions. Experiments validate the achievement of exceptional performance on established benchmarks, concurrently showcasing remarkable efficiency during both training and testing intervals.

Two-stream models have achieved remarkable performance in various computer vision tasks. It has been widely used in action recognition, outperforming previous methods on benchmark datasets such as UCF101 and HMDB51 [28]. Moreover, the two-stream model has also found applications in other domains such as gesture recognition, video captioning, and video segmentation, demonstrating its versatility and effectiveness. Future research directions may focus on developing more efficient architectures, exploring attention mechanisms, and using unsupervised or weakly supervised learning paradigms to further build up the performance and generalization capabilities of two-stream models.

### 2.3. SlowFast Networks

During the preceding years, an array of video action recognition networks has been put forth by researchers, including 2D CNN, 3D CNN, and I3D network. However, these methods have certain limitations when dealing with challenging scenarios such as long-term dependencies and fast actions. The SlowFast network as shown in Figure 1c addresses the problem of spatio-temporal scale differences in videos by introducing two branches, slow and fast [29,30]. The slow branch is used to process low-frequency information to capture long-term timing dependencies by reducing the frame rate of the input video. The fast branch is used to process high-frequency information to capture instantaneous actions by preserving the high frame rate of the input video. This design can effectively balance information on both temporal and spatial scales. It primarily comprises two main components: the slow path and the fast path [31]. The slow path is processed at a lower frame rate, typically 1/8 or 1/16 of the input video. The fast path is processed at native framerate. The two paths extract feature representations, βC and C, at different scales, respectively, and integrate information through the fusion module. Finally, after global average pooling and classification layers, βT and T, the network outputs the behavior category of the video. The SlowFast network achieves significant performance gains on video action recognition tasks [32]. Compared with the traditional 2D CNN network and 3D CNN network, the SlowFast network can better handle long-term dependencies and fast actions, and improve the accuracy and robustness of behavior recognition. In addition, the SlowFast network structure is simple and efficient, with low computing and storage overhead, and is suitable for training and reasoning on large-scale video data [33,34].

## 3. System Model and Problem Formulation

In the context of viewing brief video content, the assessment of the video’s substance based solely on subtitles is not universally definitive. Particularly for elements devoid of auditory components, visual data assume an integral role. Consequently, a proposition emerges wherein visual attributes are incorporated within each subtitle segment to prognosticate video content. The crux of this approach pertains to the harmonious alignment of features originating from video frames and subtitle text. Subsequently, a process of multi-classification ensues, conducted upon the act of mapping subtitle spans into an equivalent vector space as their corresponding video frames. For the text mode, we input the subtitle text into the BERT pre-training model, and obtain the text features by fine-tuning the parameters. The best results across various tasks within the field of natural language processing (NLP) have been achieved by the BERT pre-training model. For the visual pattern, we extract raw frames from the video by down-sampling. Then, we use the Timesformer feature extraction method to obtain visual features, which reaches state-of-the-art results on several large datasets. We perform contextual query concatenation to jointly adjust textual and visual features for the final multi-class prediction.

**Figure 2:**
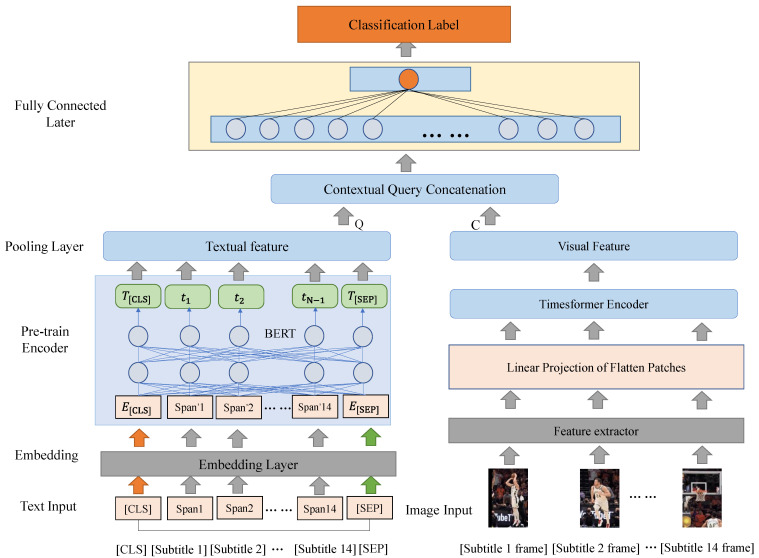
Two-channel classification framework based on cross-modal fusion.

In this chapter, an elaborate exposition is provided regarding the method introduced for the task of video classification. Since the approach of pre-training language has the capacity to augment the performance of semantic representation for textual subtitle queries, we designed a two-channel cross-modal fusion video classification method, and the process framework is shown in Figure 2 specifically.

During the observation of a video, the evaluation of its content based on subtitle text does not invariably constitute the sole criterion. Particularly for non-verbal components, the visual information assumes paramount importance. Therefore, for each subtitle span, we can add visual features to predict video content. As illustrated in Figure 2, an intricate cross-modal video classification model is devised. Specifically, we focus on the feature joint alignment of video frames and subtitle text. Following this alignment, the classification of videos is executed subsequent to the transformation of subtitle spans and their corresponding video frames into a unified vector space. For the textual modality, the subtitle text is introduced to a pre-trained language model to derive textual attributes. On the other hand, for the visual modality, the raw frames undergo down-sampling, with keyframes being captured at regular intervals within each video. The subsequent procedure involves the utilization of an attention mechanism to obtain visual attributes. The integration of contextual query concatenation facilitates the synergistic alignment of textual attributes (Q) and visual attributes (C), culminating in the ultimate prediction for video classification.

BERT and Timesformer have demonstrated outstanding performance in extracting text and visual features. They are capable of generating high-quality feature representations for text and images, respectively. Therefore, utilizing their output vectors can provide a powerful feature basis for video classification. At the same time, end-to-end error updates can require significant computing resources and time, while using only the output vectors of BERT and Vision Timesformer can significantly reduce computing costs. This is particularly advantageous for large-scale video classification tasks when resources are limited or efficient processing is required. In conclusion, considering only the output vectors from BERT and Vision Timesformer to find a joint feature space is feasible. This approach can provide high-quality feature representations and reduce computational costs.

### 3.1. Visual Feature Extraction

As deep learning continues to evolve, the architecture of the neural network exhibits as more diversified, the network structure is more complex, its feature expression ability becomes stronger and stronger, and it can learn image and video features very well.

At present, there are two main ways to extract visual features. One is to directly use the 3D convolutional network to extract the features of the entire video. The other is that overall video features are formed by feature aggregation. Due to 3D convolution, compared with 2D convolution, it adds one dimension (time dimension) to the input and then directly expands 2D convolution to 3D convolution. Although it can capture the time information of the video, at the same time it increases the parameters of the network, resulting in a larger amount of calculation, which is not conducive to real-time feature extraction. As the length of the video increases, its calculation speed will become slower and slower. Based on the above considerations, we use the method of selecting and extracting key frame features to extract video features.

Whether the selection of key frames is reasonable or not directly affects the accuracy of classification tasks. The K-means clustering algorithm is the most commonly used key frame extraction algorithm based on clustering, which has the advantages of simplicity and fast convergence speed. However, because the K-means clustering algorithm is very sensitive to the initial parameters, it is easy to fall into a local optimal solution. This paper proposes an improved hierarchical clustering algorithm based on it. This method mainly uses the characteristics of image information entropy to measure the similarity of two frames. If the similarity reaches a certain value, they will be merged into the same cluster and the extracted cluster center is used as the initial clustering result. Subsequently, the K-means algorithm is used to optimize the initial clustering result to obtain key frames.

**Figure 3:**
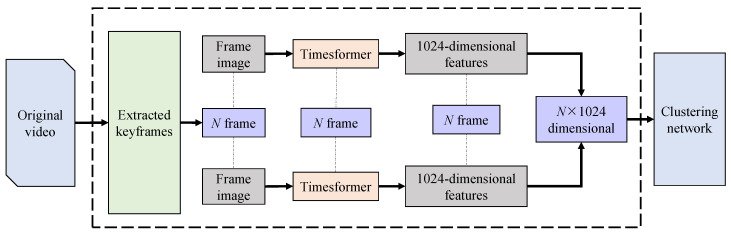
Video feature extraction process.

Figure 3 is the overall frame diagram of the visual feature extraction in this experiment. The video data have the characteristics of different time lengths. This paper mainly studies the classification of short videos. At present, the videos in various application platforms are basically edited and contain information. More parts can increase the number of views of the video. Most of the video data selected in this article are about 5 s long clips, and frame images are extracted by *ffmpeg* every second. In the process of data preprocessing, for videos whose original video data length is less than 5 s, that is, the video frame is less than 300 frames, we adopt the method of filling 0 to keep it consistent. For an original video data length greater than 5 s, that is, the video frame is greater than 300 frames, we use truncation processing.

**Figure 4:**
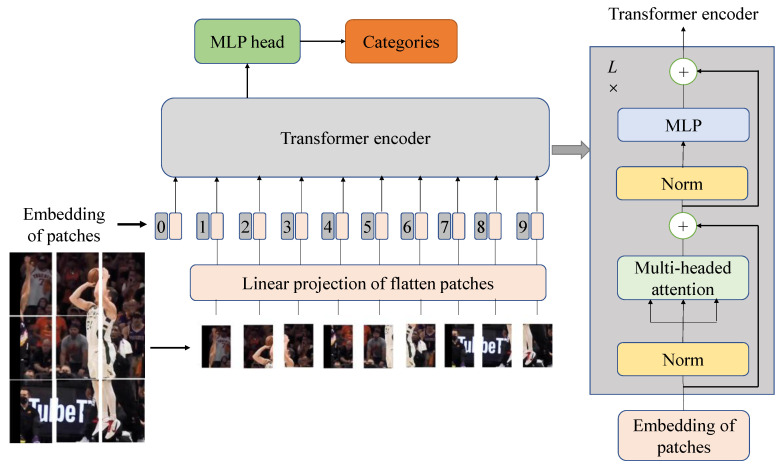
The Timesformer coding flow chart.

After the video is processed by the frame extraction operation, multiple pictures can be obtained, and then feature extraction needs to be performed on the images. At present, image features with generalization ability are widely used. At present, the commonly used convolutional neural networks for extracting image features mainly include the I3D network, SlowFast network, etc. 2D and 3D convolution are still the core algorithms for spatio-temporal features across different tasks. However, the convolutional structure has translation invariance and cannot link the image context information well, so we choose the Timesformer network model based on the attention mechanism. Since videos and sentences are both continuous, coupled with the intrinsic nature of word comprehension, which often necessitates contextual referencing within the sentence, an inclination arises to combine a certain frame of action in a short video with the rest. To completely disambiguate, the choice of a self-attention model is also completely effective for video modeling, and its structure is shown in Figure 4.

The Timesformer model stands as a video-based structure crafted exclusively upon the foundation of self-attention mechanisms. The VIT image model is adapted for video classification in the way of expanding the self-attention mechanism from its original image-based realm to spatio-temporal 3D volumes. When the VIT model has enough data for pre-training, the performance of VIT will exceed that of CNN, breaking through the limitation of the Transformer’s lack of inductive bias, and a better migration effect in downstream tasks. In the context of the Timesformer model, the perception of video occurs through the lens of a sequential compilation of patches, each drawn collectively from distinct frames. Similar to VIT, the transformation of each sequence of patches undergoes linear mapping within an embedding layer, which is further enriched with positional particulars, and then each sequence is projected into a fixed-length vector and sent to the Transformer for subsequent encoder operations.

(1) Step 1: Clip input. The input to the Timesformer model consists of a clip comprising F RGB frames of size H×W sampled from the initial video input.

(2) Step 2: Break down into patches. Following the VIT method, each frame is divided into *N* non-overlapping patches, each with dimensions P×P, in a manner where these *N* patches span the entire frame; that is, $N=HW/P^{2}$. These patches are flattened into vector $x_{\left(p,t\right)}\in {\mathbb{R}}^{3P^{2}}$, where p=1,⋯,N represents the spatial position; t=1,⋯,F represents an index on a frame.

(3) Step 3: Linear embedding. Each patch $x_{\left(p,t\right)}$ is linearly mapped to a vector $z_{\left(p,t\right)}^{\left(0\right)}\in {\mathbb{R}}^{D}$ by a learnable matrix $E\in {\mathbb{R}}^{D\times {3P}^{2}}$:

(1) $$z_{\left(p,t\right)}^{\left(0\right)}=E\cdot x_{\left(p,t\right)}+e_{\left(p,t\right)}^{pos}$$

where $e_{\left(p,t\right)}^{pos}\in {\mathbb{R}}^{D}$ denotes a location embedding that is subject to learning, and this embedding serves to encode the spatio-temporal coordinates of each individual patch. When p=1,⋯,N and t=1,⋯,F, the obtained embedding vector $z_{\left(p,t\right)}^{\left(0\right)}$ is sent to the Transformer as the input, and its function is similar to the embedded word sequence of the input text converter in natural language processing. This paper adds a special learnable vector $z_{\left(0,0\right)}^{\left(0\right)}\in {\mathbb{R}}^{D}$ at the first position of the sequence to represent the embedded classification label.

(4) Step 4: Query (*q*)—key (*k*)—value (*v*) computation. The Transformer architecture employed within this paper encompasses *L* encoding blocks as shown in Figure 4. At each block ℓ, a vector value of (q,k,v) is computed from the representation $z_{\left(p,t\right)}^{\left(\ell -1\right)}$ encoded in the previous block.

(2) $$q_{\left(p,t\right)}^{\left(\ell ,\alpha \right)}=W_{Q}^{\left(\ell ,\alpha \right)}{\mathrm{LN}(z}_{\left(p,t\right)}^{\left(\ell -1\right)})\in {\mathbb{R}}^{D_{h}}$$

(3) $$k_{\left(p,t\right)}^{\left(\ell ,\alpha \right)}=W_{K}^{\left(\ell ,\alpha \right)}{\mathrm{LN}(z}_{\left(p,t\right)}^{\left(\ell -1\right)})\in {\mathbb{R}}^{D_{h}}$$

(4) $$v_{\left(p,t\right)}^{\left(\ell ,\alpha \right)}=W_{V}^{\left(\ell ,\alpha \right)}{\mathrm{LN}(z}_{\left(p,t\right)}^{\left(\ell -1\right)})\in {\mathbb{R}}^{D_{h}}$$

where *W* represents the weight vector, LN(·) represents LayerNorm, α=1,⋯,A signifies an index corresponding to various attention heads, and A signifies the aggregate number of attention heads. Each attention head possesses a latent dimension set at $D_{h}=D/A$.

(5) Step 5: Self-attention calculation. The computation of self-attention weights is achieved through the dot product operation. The self-attention weight $\alpha_{\left(p,t\right)}^{\left(\ell ,\alpha \right)}\in {\mathbb{R}}^{NF+1}$ of query block (p,t) is obtained by the following equation:

(5) $$\alpha_{\left(p,t\right)}^{\left(\ell ,\alpha \right)}=\mathrm{SM}\left(\frac{q_{\left(p,t\right)}^{{\left(\ell ,\alpha \right)}^{T}}}{\sqrt{D_{h}}}\cdot \left[k_{\left(0,0\right)}^{\left(\ell ,\alpha \right)}{\left\{k_{\left(p^{\prime },t^{\prime }\right)}^{\left(\ell ,\alpha \right)}\right\}}_{\begin{array}{l} p^{\prime }=1,\cdots ,N\\ t^{\prime }=1,\cdots ,F \end{array}}\right]\right)$$

where SM(·) signifies the activation function known as Softmax. When attention computation is confined to a dimension, such as exclusively in time or space, it culminates in substantial computational reduction. For instance, in spatial attention, the number of query key-value pair comparisons stands at only *N* + 1, wherein unique keys reference the same frame.

(6) $$\alpha_{\left(p,t\right)}^{(\ell ,\alpha )space}=\mathrm{SM}\left(\frac{q_{\left(p,t\right)}^{{\left(\ell ,\alpha \right)}^{T}}}{\sqrt{D_{h}}}\cdot \left[k_{\left(0,0\right)}^{\left(\ell ,\alpha \right)}{\left\{k_{\left(p^{\prime },t\right)}^{\left(\ell ,\alpha \right)}\right\}}_{p^{\prime }=1,\ldots ,N}\right]\right)$$

(6) Step 6: Coding. The encoding $z_{\left(p,t\right)}^{\left(\ell \right)}$ in block ℓ is obtained by weighting the vector of values computed by the self-attention system of each attention head.

(7) $$s_{\left(p,t\right)}^{\left(\ell ,\alpha \right)}=\alpha_{\left(p,t),(0,0\right)}^{\left(\ell ,\alpha \right)}v_{\left(0,0\right)}^{\left(\ell ,\alpha \right)}+\sum_{p^{\prime }=1}^{N}\sum_{t^{\prime }=1}^{F}\alpha_{\left(p,t),(p^{\prime },t^{\prime }\right)}^{\left(\ell ,\alpha \right)}v_{\left(p^{\prime },t^{\prime }\right)}^{\left(\ell ,\alpha \right)}$$

Subsequently, these vectors from all attention heads are subjected to projection and directed through an MLP layer, utilizing the residual connections in each operation.

(8) $${z^{\prime }}_{\left(p,t\right)}^{\left(\ell \right)}=Wo\left[\overset{s_{\left(p,t\right)}^{\left(\ell ,1\right)}}{\underset{s_{\left(p,t\right)}^{\left(\ell ,A\right)}}{\vdots }}\right]+z_{\left(p,t\right)}^{\left(\ell -1\right)}$$

(9) $$z_{\left(p,t\right)}^{\left(\ell \right)}=\mathrm{MLP}(\mathrm{LN}({z^{\prime }}_{\left(p,t\right)}^{\left(\ell \right)}))+{z^{\prime }}_{\left(p,t\right)}^{\left(\ell \right)}$$

(7) Step 7: Categorical embedding. The final clip embeddings are obtained from the class-labeled final block.

(10) $$y=\mathrm{LN}(z_{\left(0,0\right)}^{\left(L\right)})\in {\mathbb{R}}^{D}$$

After being processed by multiple Transformer encoder layers, the output of the model’s last position is considered as a representation of the entire image.

### 3.2. Text Feature Extraction

**Figure 5:**
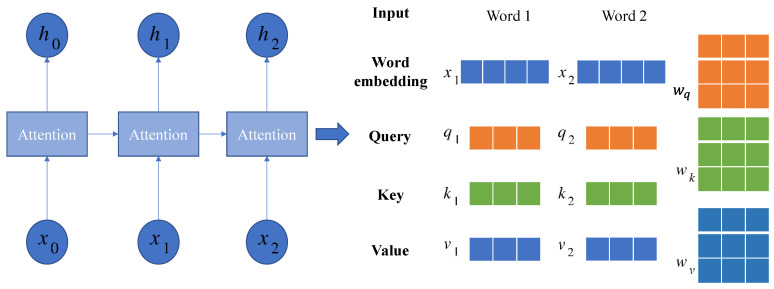
Word vector model.

In neural machine translation, the Seq2Seq model is a widely used architecture. Typically, a Seq2Seq model consists of two recurrent neural networks (RNNS) for processing sequential data. However, such a model suffers from the obvious limitation of not being able to perform parallel computations, as it requires processing each element of the sequence in turn. The word vector model tool, Word2vec, can efficiently train on millions of dictionaries and huge datasets, and use the word vectors obtained by it to effectively determine the similarity between different words [10]. The Word2vec model is based on two algorithms, Skip-Gram and CBOW. The former predicts the surrounding context word by giving the target word, and the latter predicts the target word by given the context of the surrounding word. One disadvantage of these algorithms is that the expression of the same word in different contexts does not change after pre-trained word vectors. To solve the above problems, this paper considers using the BERT word vector model to replace the sequence model and the Word2vec word vector model, as shown in Figure 5, where $x_{0},\;x_{1}$ and $x_{2}$ are word embeddings of different words, $h_{0},\;h_{1}$ and h are the content streams after passing through the attention network, $\left(q_{1},\;k_{1},\;v_{1}\right)$ and $\left(q_{2},\;k_{2},\;v_{2}\right)$ are the query—key—value vector values, and $w_{q},\;w_{k}$ and $w_{v}$ represent the weight vectors.

Language model pre-training has demonstrated its effectiveness in enhancing a spectrum of natural language processing tasks encompassing a natural language inference. Presently, two strategies underpin the application of pre-trained language models to downstream tasks: feature-based and fine-tuning-based. In the feature-based approach, exemplified by ELMo [35], task-specific architectures are enhanced with supplementary pre-trained representations as additional attributes. Conversely, fine-tuning-based methods, typified by a generative pre-training Transformer (OpenAI GPT) [36], incorporate minimal task-specific parameters. These models then undergo training on downstream tasks through a straightforward fine-tuning of all pre-trained parameters.

While pursuing distinct methodologies, both strategies share the same pre-training objective function, utilizing a singular-term language model to acquire a universally applicable language representation. The text feature extraction framework is shown in Figure 5.

**Figure 6:**
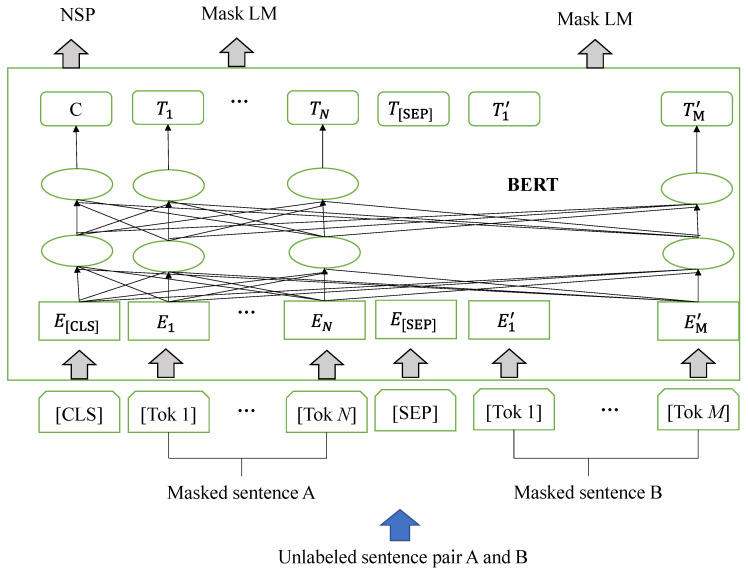
Text feature extraction framework.

It is contended that the prevailing techniques impose constraints on the potential of pre-trained representations, particularly in the context of fine-tuning based methods. The primary constraint stems from the fact that conventional language models adhere to a unidirectional nature, thereby constraining the available options for pre-training architectures. These limitations are not optimal for sentence-level tasks, which ignore the incorporation of contexts from different directions of the sentence. The BERT can be used to extract text features. Its framework has two steps: pre-training and fine-tuning based methods. In this paper, we use the fine-tuning-based method in BERT as shown in Figure 6.

The input text type of this article is subtitle information, which consists of a group of sentence pairs A and B. Firstly, word segmentation is performed on the sentence pair, and a piece of text is divided into *N* or *M* individual words or sub-words. The input length is fixed to 512. If the length of the input text is greater than 512, it truncates the input text, and if the length of the input text is insufficient, it takes a special symbol to fill. A special tag [CLS] is added at the beginning of the input text to indicate that the text belongs to a classification task, and [SEP] tags are used to indicate the segmentation between sentences. The BERT model optimizes its weight through multiple rounds of pre-training iterations. Finally, each input tag corresponds to a 1024-dimensional vector denoted by *E* or *E′*. These vectors constitute the hidden state representations *T* and *T′* of the last layer and can be used as feature representations, masked language modeling (LM) and next sentence prediction (NSP), for downstream tasks. 

### 3.3. Cross-Modal Feature Fusion Framework

In this paper, cross-modal fusion techniques are introduced to fuse information from different modalities together to address the problem of multimodal information processing and analysis. In the fusion process, early fusion will inhibit the links within or between modalities, resulting in the loss of video semantics, and the interaction between different modalities cannot be achieved. Therefore, this paper adopts the late fusion method, which inputs each modality information into the clustering network and uses the dot product operation to obtain the final video feature vector. This late fusion way helps to preserve the richness of multimodal information and realizes the interaction between different modalities, which improves the model performance.

After the visual features and text features of video frame-level images are extracted by Timesformer and BERT, the frame-level features need to be aggregated to obtain video-level features before video classification. Previously, the long short-term memory network LSTM and gated recurrent unit (GRU) can obtain the timing information of the video. However, the next vector of the locally aggregated descriptors (NextVLAD) network and the AttentionCluster network, which are conducive to scene recognition, are more effective for aggregating visual features and text features.

The NextVLAD network reduces the overall parameters of the model by reducing the input dimension and splitting it into multiple groups. It first increases the dimension of y to obtain $\dot{y}$, and then divides $\dot{y}$ into groups to obtain $\tilde{y}$, and then calculates the weights with the cluster centers, respectively. Finally, the global features are aggregated by grouping results. Assume that the video has M frames, and the feature description y of each frame is N-dimensional. For the K cluster centers included, NetVLAD first encodes the features of each frame into an N×K feature vector, as shown in Equation (11).

(11) $$\begin{array}{l} v_{ijk}{}^{g}=\alpha_{g}({\dot{y}}_{i})\alpha_{gk}({\dot{y}}_{i})({\tilde{y}}_{ij}{}^{g}-c_{kj})\\ g\in \{1,\ldots ,G\},i\in \{1,\ldots ,M\},k\in \{1,\ldots ,K\} \end{array}$$

where $c_{k}$ is the N-dimensional eigenvector coordinates of the cluster center k, G is the number of groups, and the similarity measure calculation equation is as follows:

(12) $$a_{gk}(\dot{y})=\frac{e^{W_{gk}^{T}{\dot{y}}_{i}+b_{gk}}}{\sum_{S=1}^{K}e^{W_{gs}^{T}{\dot{y}}_{i}}+b_{gs}}$$

(13) $$\alpha_{g}({\dot{y}}_{i})=\sigma (w_{g}^{T}{\dot{y}}_{i}+b_{g})$$

where σ is the sigmoid function, $\alpha_{g}({\dot{y}}_{i})$ computes attention weights for all groups.

The encoding feature l of the entire video is expressed as follows:

(14) $$l_{jk}=\sum_{i,j}v_{ijk}^{g}$$

NextVLAD divides video features into multiple groups for clustering operations, and introduces an attention mechanism to add weights to different groups. It uses AttentionCluster attention clustering while adding offset operations, thereby increasing the weight of frames strongly related to tags in video content. Finally, several local features are aggregated into a video global feature.

## 4. Experimental Results and Discussion

To realize the short video classification task, we extract its text information features and visual information features from the video. In our experiments, the textual attributes obtained from videos encompass elements such as video titles, subtitle information, and descriptions of videos. We stem these textual features and remove stop words using the standard BERT contextual attention mechanism. We use the filtering mechanism to perform a zero-fill operation for those that are not long enough, and directly truncate those that are too long to remove interference [37,38]. For the visual features of the video, we use the Timesformer method to extract video features using the spatio-temporal self-attention mechanism. First, the model obtained by the Timesformer network pre-trained on the ImageNet dataset is used to extract the features of each image. The 1024-dimensional vector obtained by the last fully connected layer of Timesformer is used as the feature of each image.

In the experiments, we use Pycharm as the development tool. Based on the Pytorch 1.13.0 deep learning framework, we use Python 3.7 as the development language. The main configuration of the computing server is as follows: (1) Operating system: Ubuntu21.04, (2) CPU: Intel(R) Core(TM) i7-11700K CPU @ 2.50 GHz, (3) Memory: 32 GB, (4) GPU: RTX2080Ti.

### 4.1. Experimental Dataset

To explore the scalability of the model, the dataset of this experiment is the BOVText dataset, which is a large-scale bilingual open video text dataset [39]. First of all, it has more than 2000 videos and more than 1,750,000 + frame fragments, which is 25 times larger than the existing largest dataset with text in videos, and the model can have a good generalization effect on it. Second, the dataset covers 31 open categories and one unknown category, with wide application options. Additionally, it contains the public dataset Kinetics-400 [40]. Kinetic stands as an extensively utilized dataset for the recognition of video actions, encompassing 400 distinct categories of human actions, with each category featuring approximately 400 video clips. These video clips are around 10 s in length and originate from real-world Internet videos. Each clip contains a single human action, such as running, jumping, cycling, etc.

### 4.2. Performance Evaluation Index

With the objective of assessing the efficacy of multimodal classification results, four prevalent metrics are introduced: Accuracy (*AC*), Precision (*PE*), Recall (*RE*), and *F*1 Score. The larger the value of these performance indicators, the better the classification effect, and their definitions are as follow equations.

(15) $$AC=\frac{TP+TN}{n}$$

(16) $$PE=\frac{TP}{TP+FP}$$

(17) $$RE=\frac{TP}{TP+FN}$$

(18) $$F1=\frac{2PE\times RE}{PE+RE}$$

where TP means that the judgment is positive and it is actually positive, TN means that it is judged as negative and it is actually negative, FP means that it is judged as positive and it is actually negative, and FN means that it is judged as negative and it is actually positive, n=TP+TN+FP+FN.

### 4.3. Experimental Results and Analysis

The experiments mainly include single-feature experiments, multi-feature experiments, and public dataset comparison experiments. The accuracy rate commonly used in video classification datasets is the Top@*k* accuracy rate. In this experiment, the classification model performance evaluation indicators use Top@1 and Top@5. The dataset in this article is divided into 32 categories, so the model will output a one-dimensional vector containing 32 values. Each value indicates the probability that the video belongs to each category, where Top@1 refers to the correct classification of the results predicted by the model, the accuracy rate when the sample proportion is the highest. Top@5 refers to the accuracy rate in the first five categories of the predicted results when the proportion of correctly classified samples is the highest in the predicted results of the model. The accuracy requirement of the latter is wider than that of the former, so the value of the latter is generally greater than that of the former. At the same time, we use F1 Score which takes Precision and Recall into account to evaluate the model performance.

(1) Single-mode feature

The single-feature experiment is an experiment in machine learning and statistical modeling that uses only one feature to train and test a model. In single-feature experiments, other features are usually considered irrelevant or ignored, because the purpose is to understand the impact of a single feature on model performance, and it is mainly used to compare the accuracy of a single network and a combined network. Based on the outcomes in Table 1, it is evident that the accuracy by the amalgamated network model surpasses that of the individual network, and the accuracy of the combined network is 1% higher than that of the single network. It can be seen that the effect of the same feature on a single network may be good or bad, but the performance of the combined network model is better than that of the single network model regardless of the feature of that modality. At the same time, comparing the impact of different features of video data on its classification task, it is found that the most critical data is visual information, and the impact of text information on classification accuracy is slightly lower than that of visual information.

(2) Cross-modal fusion

The cross-modal fusion experiment refers to the fusion of the video-level features of the data of multiple modalities in the video through the clustering network each time, and then input them into a single network or combined network for classification. This experiment is mainly used for comparing the classification performance between a single modality and a fusion of two modalities. It can be seen from Table 2 that the effect of combining the features of video visual information and text information into joint features as video features for video classification is better than that of any single-modal feature, and the accuracy of the combined network model is the best. Compared with the accuracy rate of a single network, the accuracy rate is increased by 2%, and the effect of the NextVlAD model is better than that of the AttentionCluster model. Compared with the single-feature experiment, the accuracy rate increased by 4% to 11%.

(3) Comparison of public datasets

Table 3 provides the comparison of the experimental results with public datasets. The results presented in Table 3 highlight the superiority of the CFVC model introduced in this paper over other existing models within the public dataset context. In comparison to the dual-stream network that also uses modality fusion, the accuracy rate is improved by more than 10%, because the Transformer-based Timesformer is used as the video feature extraction network. For the VIT-L model trained end-to-end [29], the accuracy rate also increased by 7.1%. To sum up, it is not difficult to see that the method proposed in this paper is superior to the current mainstream convolutional neural network-based method, because they use different structures for feature extraction, and it also proves that the Transformer-based model extraction ability is better than CNN. Although CNN has advantages in extracting low-level features and structures, how to associate with high-level semantic information is a difficult problem, and Transformer uses the attention mechanism to capture global context information to increase their relevance.

## 5. Conclusions

In this paper, we first study the video features of different modalities and the adopted feature extraction methods. According to the information characteristics of each video modality, different network models are used to extract the corresponding features, so that it can represent the information of the modality well. Then, through the clustering algorithm, the features of the two modalities are fused to obtain the features of the video, thereby improving the representation of the overall features. The final features are made more useful for classification tasks by means of modality fusion. In experiments, classification evaluation is performed on our dataset. The comparison experiment mainly studies the difference between our model and different models. The experiment results show that visual information has the greatest impact on video classification tasks, and the accuracy of the model can be effectively improved by modality fusion, thus improving the accuracy of classification. Correlative results reveal the effectiveness of our model, which has certain advantages over other models. Due to the limited types and number of videos in the training dataset, the impact of different training datasets on the classification performance has not been further investigated. In future work, we will try to expand the type and number of videos to improve the classification performance.

## Figures and Tables

**Table 1:** Experimental results of single mode.

Mode	Feature	Top@1 (%)	Top@5 (%)	*F*1 (%)
NextVLAD	Video frame	60.1	70.9	65.3
NextVLAD	Subtitle	55.9	63.2	58.7
AttentionCluster	Video frame	58.2	69.4	63.3
AttentionCluster	Subtitle	52.0	62.3	56.2
NextVLAD-AttentionCluster	Video frame	61.1	79.0	67.9
NextVLAD-AttentionCluster	Subtitle	57.3	63.1	59.4

**Table 2:** Experimental results of cross-modal fusion.

Mode	Feature	Top@1 (%)	Top@5 (%)	*F*1 (%)
NextVLAD	Video frame and Subtitle	64.3	72.8	68.2
AttentionCluster	Video frame and Subtitle	63.2	71.2	65.9
NextVLAD-AttentionCluster	Video frame and Subtitle	65.8	82.2	73.2

**Table 3:** Comparison results with other methods.

Mode	Feature	Top@1 (%)	Top@5 (%)	*F*1 (%)
I3D [23]	Video	70.1	90.1	78.1
R [2 + 1]D-Two-Stream [27]	Video + stream	73.6	90.5	81.1
Two-Stream I3D [28]	Video + stream	75.7	92.6	83.3
SlowFast [29]	Video	77.4	93.2	84.5
VIT-L (64 frames) [34]	Video	80.5	94.5	86.9
CFVC (Our method)	Video + text	87.6	96.3	91.7

## Data Availability

The authors confirm that the data supporting the findings of this study are available within the article.
